# Programmed Cell Death in Cancer

**DOI:** 10.1002/mco2.70357

**Published:** 2025-08-31

**Authors:** Yuang Wei, William Hankey, Dongliang Xu, Fuwen Yuan

**Affiliations:** ^1^ Cancer Research Center School of Integrative Medicine, Shanghai University of Traditional Chinese Medicine Shanghai China; ^2^ Urology Centre Shuguang Hospital Affiliated to Shanghai University of Traditional Chinese Medicine Shanghai China; ^3^ Surgical Institute of Integrative Medicine, Shuguang Hospital Affiliated to Shanghai University of Traditional Chinese Medicine Shanghai China; ^4^ Shanghai Key Laboratory of Traditional Chinese Clinical Medicine Shanghai China; ^5^ Department of Genetics The University of North Carolina At Chapel Hill Chapel Hill North Carolina USA

**Keywords:** anticancer therapy, cancer, ferroptosis, programmed cell death, prostate cancer

## Abstract

Cancer remains the most lethal disease globally, despite the significant progress made in early screening, surgery, and therapeutic development in recent decades. Programmed cell death (PCD) is a genetically regulated process essential for eliminating aberrant cells, yet its dysregulation drives tumorigenesis and therapy resistance. In this review, we present a complete discovery timeline of them and comprehensively synthesize the roles and mechanisms of major PCD forms, such as apoptosis, necroptosis, autophagy, pyroptosis, ferroptosis, and cuproptosis, across diverse cancer types. We not only detail the molecular mechanisms, dual functions, and alterations of these PCD modalities in cancers, but also summarize their interconnections and intrinsic crosstalk. Furthermore, we comprehensively discuss how diverse therapies, including chemotherapy, radiotherapy, immunotherapy, targeted agents, and hormone therapy, engage and manipulate specific PCD pathways, revealing the involvement of PCD in cancer treatment mechanisms. This review integrates extensive preclinical and clinical evidence on PCD‐targeted therapies with an in‐depth focus on ferroptosis, including its regulatory networks and therapeutic relevance. Special emphasis is placed on prostate cancer, highlighting the PCD‐based translational opportunities in this common malignancy. Taken together, we provide novel insights into the complex interplay between PCD and cancer biology and offer a framework for developing precision oncology therapies.

## Graphical abstract

Cancer is one of the most common and lethal diseases globally, with programmed cell death (PCD) playing a pivotal role in its pathogenesis and treatment. This comprehensive review covers the origin, developmental trajectory, and mechanisms of various PCDs, as well as their molecular connections with cancers. Latest studies have increasingly highlighted the significance of PCD dysregulation in cancer, as well as its biological interconnection and molecular crosstalk. Moreover, dominant anticancer therapies (e.g., chemotherapy, targeted therapy, radiotherapy) and alternative treatments (e.g., natural products, traditional medicine) have shown relations to PCDs, making it an urgent need to explore the potential of PCD‐targeted treatments in cancers. To conclude, this review provides a comprehensive perspective on PCDs in cancer initiation, progression, and response to therapy.

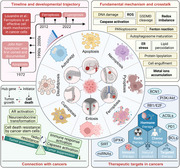

## 1 Introduction

Cell death is categorized into accidental and programmed forms. Accidental death results from physical, chemical, or mechanical disruptions, while programmed cell death (PCD) is a genetically regulated process triggered under specific cellular conditions [1]. The 2018 update by the Nomenclature Committee on Cell Death classified 12 distinct PCD mechanisms, ranging from classical apoptosis and pyroptosis to newer forms like ferroptosis. Each subtype is characterized by unique biochemical signatures and physiological implications, offering nuanced insights into disease pathogenesis and therapy [2].

With the deepening understanding of the regulatory mechanisms underlying cell death, emerging PCD modalities have been progressively studied, such as copper (Cu) overload‐induced cuproptosis [3], sodium‐associated necrotic cell death under sodium overload [4], and disulfide stress‐triggered disulfidptosis [5]. These emerging pathways underscore the complexity of intracellular stress responses and highlight previously unrecognized links between metal ion homeostasis, metabolic stress, and cell viability. As a result, the study of PCD has evolved into a central pillar of biomedical research, with direct relevance to cancer biology, immunology, and precision medicine.

The dysregulation of PCD is now recognized as a hallmark of cancer and other pathological states. In oncology, PCD exhibits a dual character: while it suppresses tumor development by eliminating unstable cells, many tumors manipulate these pathways to form immune evasion and facilitate metastasis and therapeutic resistance [6]. Pan‐cancer analyses have revealed recurrent mutations and expression patterns in PCD‐related genes, with significant correlations to prognosis and treatment responses. These findings have prompted the development of targeted therapies aimed at modulating cell death pathways to improve clinical outcomes [7, 8].

Leveraging PCD has become a therapeutic strategy in cancers, and treatment approaches have progressively shifted from isolated pathway modulation toward integrated strategies. For example, the activation of PANoptosis, a form of PCD which combines apoptosis, necroptosis, and pyroptosis via the assembly of their core components into a multiprotein complex known as the PANoptosome, represents a promising multitargeted strategy for cancer therapy. Emerging studies suggest this may be more immunogenic and effective at overcoming resistance mechanisms [9, 10, 11, 12]. Therapies designed to activate PANoptosis have shown potential in preclinical models and are under exploration for refractory tumors such as triple‐negative breast cancer (TNBC), a refractory subtype of breast cancer (BCa) that lacks expression of estrogen receptor (ER), progesterone receptor, and human epidermal growth factor receptor 2 (HER2) [13, 14]. In recent years, ferroptosis, due to its dependence on iron (Fe) metabolism and lipid peroxidation, has gained attention for its capacity to reshape the tumor microenvironment (TME) and enhance responses to immunotherapy [15, 16, 17]. Besides, clinical translation has witnessed breakthroughs of multimodal strategies based on PCD‐targeted treatment. A hallmark example of successful clinical translation is the approval of programmed cell death protein 1/ligand 1 (PD‐1/PD‐L1) immune checkpoint inhibitors (ICIs), such as pembrolizumab and nivolumab [18]. These therapies reactivate cytotoxic T lymphocytes and restore apoptosis in tumor cells, demonstrating clinical benefits across various cancers and have become standard‐of‐care treatments in multiple oncologic guidelines [19]. Additionally, ferroptosis inducers are moving toward clinical application, offering promise for overcoming apoptosis resistance in refractory cancers [19]. These therapies offer an avenue to overcome resistance to apoptotic cell death in tumors.

Despite these advances, several challenges remain. The mechanisms of PCD resistance in therapy‐resistant tumors are not fully understood, and the complex interplay between PCD types within the TME demands deeper investigation [20]. This review is structured to first examine the molecular underpinnings and classification of PCD, then transition into its role in cancer development and therapy, following a discussion on translational challenges and future research directions. We aim to synthesize current insights from pan‐cancer analyses on PCD, spotlight emerging therapeutic strategies, especially ferroptosis and prostate cancer (PCa), and provide a comprehensive theoretical framework for advancing precision oncology.

## 2 Roles of PCD in Cancers

**FIGURE 1:**
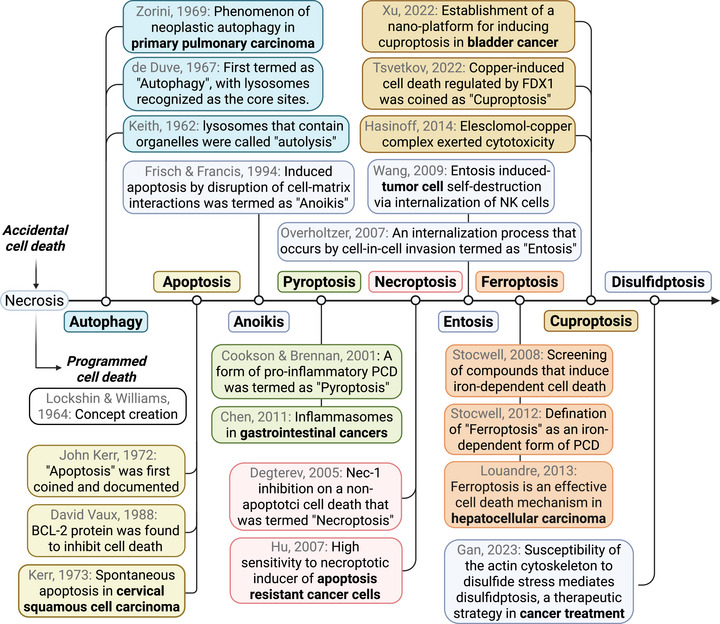
The milestones of major PCDs and their pioneering studies in cancers. The classification of cell death underwent a paradigm shift in the 1960s, transitioning from the traditional view of accidental cell death (necrosis) to the recognition of regulated physiological cell death mechanisms. We outlined the landmark events of major PCDs using different colors, including the proposal of their concepts, the discoverers, and the first research reported in cancer.

Many subtypes of PCD have been well documented in the pathogenesis and treatment of cancers. In addition to the best‐known forms of PCD like apoptosis, autophagy, necroptosis, pyroptosis, and ferroptosis [21, 22], studies are increasingly uncovering the involvement of other types of PCD in cancers [23], including entosis [24], pyroptosis [25], parthanatos [26], and the newly identified cuproptosis and disulfidptosis [27, 28] (Figure 1). In this section, we will provide an overview of the recent updates on major PCD types and the molecular networks involved in cancer progression and treatment.

### 2.1 Apoptosis: The Fundamental Programmed Cell Death

Apoptosis, the first formally recognized form of PCD in mammalian cells, is a tightly regulated and evolutionarily conserved process crucial for maintaining cellular homeostasis. The term “apoptosis” was introduced in 1972 by Kerr, Wyllie, and Currie, drawing inspiration from James Cormack at the University of Aberdeen [29]. Morphologically, apoptosis is characterized by distinctive features such as cytoplasmic shrinkage, chromatin condensation, and the formation of apoptotic bodies, phenomena observed across both physiological and pathological conditions [30]. Mechanistically, apoptosis is broadly categorized into two pathways: the intrinsic (mitochondrial) and extrinsic (death receptor‐mediated) pathways, each governed by distinct molecular triggers and signaling cascades. The intrinsic pathway is typically initiated by intracellular stress signals, including DNA damage, cellular stress, and mitochondrial dysfunction. It is primarily regulated by the B cell lymphoma‐2 (BCL‐2) family, which contains antiapoptotic proteins (e.g., BCL‐2, BCL‐XL, and A1/BFL1), proapoptotic BCL‐2 homology region 3 (BH3)‐only proteins (e.g., BIM, BID, and NOXA), and proapoptotic effectors such as BCL‐2‐associated X (BAX), BCL‐2 antagonist/killer 1, and BCL‐2‐related ovarian killer. Upon activation, these effectors mediate mitochondrial outer membrane permeabilization (MOMP), leading to cytochrome c release and the activation of caspase‐9, which in turn triggers the downstream effector caspases, such as caspase‐3 and caspase‐7, culminating in cell death [9]. The extrinsic pathway, on the other hand, is initiated by the engagement of membrane‐bound death receptors, such as Fas (CD95), tumor necrosis factor receptor 1 (TNFR1), and TRAIL receptors (DR4 and DR5). These receptors possess a cytoplasmic death domain (DD) that enables the recruitment of adaptor proteins. For instance, upon binding of Fas ligand (FasL) secreted by activated T cells to Fas, the adaptor protein Fas ‐ associated DD (FADD) is recruited, which subsequently activates initiator caspase‐8. This leads to a caspase cascade that converges on effector caspases, executing apoptosis in a controlled and immunologically silent manner [6].

In the context of diseases, Kerr and Searle [31] first observed a pronounced epithelial cell loss in the prostates of castrated rats, an effect attributed to heightened apoptotic activity, which was mitigated by exogenous testosterone administration, thereby preserving cell integrity postcastration. At the same time, they first reported the spontaneous apoptosis in squamous carcinomas of the uterine cervix [32]. The research interest in apoptosis in cancer experienced a significant surge starting after the 1990s. The BCL‐2 family, with its pro‐ and antiapoptotic constituents, is implicated in the delicate balance of cell survival signaling, often perturbed in cancers through the overexpression of antiapoptotic proteins, fostering a survival bias and impeding therapeutic efficacy. Elevated BCL‑2 expression has been correlated with poor prognosis and resistance to hormone therapy, targeted agents, or cytotoxic chemotherapy across a broad spectrum of malignancies, such as lymphomas, BCa, and non‑small cell lung cancer [33, 34, 35]. Recent studies have further emphasized that restoration of PTEN function combined with radiotherapy (RT), as well as dual inhibition of the PI3K/Akt signaling axis and antiapoptotic BCL‑XL in PTEN‑deficient tumors, can effectively overcome BCL‑2‐mediated survival advantages, highlighting the multifaceted regulation of apoptotic pathways in diverse cancer contexts [36, 37, 38]. High‐grade intraepithelial neoplasia is recognized as a critical precursor in the neoplastic development of vaginal, anal, cervical, pancreatic, colorectal, gastric, and prostatic cancers, characterized by cell proliferation and an augmented proliferation index [39]. The aberrant accumulation of these neoplastic cells suggests a disruption in the regulatory pathways governing cell proliferation and death [40]. Johnson et al. [41] have demonstrated that BCL‐2 expression is significantly upregulated in high‐grade prostatic intraepithelial neoplasia, compared with PCa and benign prostatic epithelium, suggesting an early involvement of apoptotic dysregulation in PCa pathogenesis. Further studies have corroborated these findings, with BCL‑2 expression detectable across multiple cellular hierarchies in diverse tumor precursor lesions. Pronounced immunoreactivity has been observed in subsets of ductal carcinoma in situ of the esophageal, anal, and prostatic metaplasia [42, 43, 44]. These observations collectively underscore the pivotal role of apoptotic dysregulation in the early stages of tumorigenesis across a broad spectrum of cancers.

Moreover, the proapoptotic effector BAX constitutes a critical control point in the intrinsic death pathway and has emerged as a promising therapeutic target in treatment‑refractory malignancies [45]. Phytochemicals such as apigenin and quercetin have been shown to shift the balance toward apoptosis by downregulating antiapoptotic BCL‑2 family members and upregulating BAX in diverse cancer cell models, such as breast and PCa cell lines [46, 47, 48]. The extrinsic apoptosis cascade, triggered by death ligands such as TRAIL and FASL, likewise plays a central role in tumor immunosurveillance and has been exploited genetically and pharmacologically across multiple cancer types [49]. Immunotherapies leveraging TRAIL/FASL signaling have demonstrated potent induction of apoptosis in solid tumors [50, 51, 52]. Collectively, these findings illustrate the molecular networks of intrinsic and extrinsic apoptotic pathways contributing to oncogenesis, tumor progression, and therapy resistance across a broad spectrum of cancers, and underscore apoptosis as a readily targetable mechanism in current and emerging cancer therapies.

### 2.2 Necroptosis: A Form of Programmed Necrosis

Necroptosis, a form of programmed necrosis distinct from apoptosis, was first characterized by Degterev et al. [53]. It is characterized by morphological features such as cell and organelle swelling, loss of membrane integrity, production of reactive oxygen species, and the release of apoptosis‐inducing factor (AIF), which do not occur in necrosis [54]. Necroptosis can be initiated in response to a range of stimuli, including death receptor signaling, pathogen invasion, and various forms of cellular stress. Notably, stimulation of death receptors such as TNFR1, Fas, DR4, and DR5 can lead to necroptosis under conditions where caspase‐8 is inhibited or absent. Under normal circumstances, caspase‐8 prevents necroptosis by cleaving receptor‐interacting protein kinase 1 (RIPK1). However, when caspase‐8 is inactive, RIPK1 becomes stabilized and can initiate downstream signaling independent of caspase activity. The identification of small molecule inhibitors like necrostatin‐1 (Nec‐1), which selectively inhibits RIPK1, has been instrumental in delineating necroptosis from other forms of cell death [55]. In a canonical RIPK1‐dependent necroptosis pathway, the activation of TNF leads to the assembly of a transient intracellular complex known as complex I, which is formed through the interaction of the TNFR1 DD with various proteins. This complex recruits several key proteins, including the adaptor protein TNF receptor‐associated DD (TRADD), RIPK1, and E3 ubiquitin ligases. Subsequently, RIPK1 undergoes polyubiquitination, which facilitates the formation of complex II, comprising FADD, caspase‐8, RIPK1, RIPK3, and mixed lineage kinase domain‐like protein (MLKL), all of which are crucial for mediating necroptosis. Notably, the inactivation of caspase‐8 is essential, as it enables RIPK1 to acquire enzymatic activity and interact with RIPK3 to form complex II. Within this complex, RIPK3 phosphorylates MLKL, ultimately triggering necroptosis by compromising plasma membrane integrity. Additionally, necroptosis can be initiated in caspase‐deficient cells through the activation of pattern‐recognition receptors (PRRs), such as Toll‐like receptors 3 and 4 (TLR3 and TLR4) and Z‐DNA‐binding protein 1 (ZBP1) [56]. The dysregulation of necroptosis has significant implications for various diseases, highlighting its potential as a therapeutic target in cancer.

In various cancers, RIPKs have emerged as key regulators of necroptosis, a programmed form of necrotic cell death with therapeutic relevance. Dysregulation of RIPK3 has been frequently observed in tumor cells, including those from colorectal, breast, and lung cancers, contributing to impaired necroptotic signaling and enhanced tumor survival [57, 58, 59]. Restoration of RIPK3 expression can reactivate the epigenetic modifications of MLKL and induce necroptosis, thereby suppressing tumor growth [60]. In some cancers, RIPK3 levels are elevated during early disease stages and inversely correlated with tumor burden and biomarkers of malignancy, suggesting a tumor‐suppressive role in the initial phases of oncogenesis [61]. Natural compounds such as Erigeron breviscapus have been shown to induce necroptosis in cancer cells by activating the RIPK1–RIPK3–MLKL signaling axis, highlighting their potential as necroptosis‐based therapeutic agents [62].

Furthermore, members of the sirtuin (SIRT) family, particularly SIRT3, have been implicated in modulating necroptotic responses in cancers. Biologically, SIRTs exert nicotinamide adenine dinucleotide (NAD)⁺‐dependent deacetylase activity on a range of substrates, including transcription factors, metabolic enzymes, and chromatin‐associated proteins, thereby influencing cellular stress responses and cell death pathways. In the context of necroptosis, SIRT3‐mediated deacetylation of mitochondrial proteins may enhance ROS production, which promotes RIPK1/RIPK3 activation [63]. Consequently, elevated SIRT levels can potentiate necrosome assembly and downstream MLKL phosphorylation. Elevated expression of these SIRTs has been associated with advanced tumor features, such as lymph node metastasis, higher histological grades, and reduced patient survival in several malignancies, including lung cancer, colorectal cancer, and PCa models [64, 65, 66]. These findings underscore the multifaceted role of necroptotic regulators like RIPKs and SIRTs in cancer biology and point toward their potential exploitation for therapeutic intervention across a broad oncologic spectrum.

Necroptosis and apoptosis, sharing some common characteristics, can be simultaneously modulated as “PANoptosis” in cancers. For instance, the loss of reticulocalbin 1 (RCN1) induces apoptosis in AR‐negative DU145 cells but promotes necroptosis in AR‐expressing LNCaP cells [67]. Nevertheless, the authors suggest that this dual effect does not occur through the regulation of AR expression or androgen levels. They reported that RCN1 simultaneously regulates apoptosis and necroptosis by modulating PTEN/Akt and CaMKII activity, respectively. It is indicated that caspase‐8 activity should be further explored based on the fundamental mechanisms underlying the occurrence of apoptosis and necroptosis. Additionally, there is growing evidence that ZBP1‐induced apoptosis may be accompanied by necroptosis, emphasizing the complex role of ZBP1 in regulating cell death in multiple cancer models [68, 69].

### 2.3 Autophagy: Cellular Recycling and Survival Strategy

Autophagy is an evolutionarily conserved catabolic process that facilitates the degradation and recycling of intracellular components, playing a fundamental role in cellular homeostasis and stress adaptation. Autophagy exists in three primary forms, chaperone‐mediated autophagy, microautophagy, and macroautophagy, characterized by distinct mechanisms of cargo delivery to lysosomes [70]. The term “autophagy,” derived from the Greek words autos (self) and phagein (to eat), captures the essence of this “self‐eating” process, meaning delivering cytoplasmic cargo to the lysosome for degradation. This cellular process degrades and recycles cytoplasmic components, including organelles and protein aggregates, through the formation of autophagosomes that subsequently fuse with lysosomes. This recycling is essential for cellular energy maintenance, and dysregulation of autophagy is implicated in various diseases, such as cancer and neurodegeneration [71, 72]. Autophagy is classified into two distinct functional categories: autophagy‐dependent cell death (ADCD) and autophagy‐mediated cell death (AMCD). ADCD refers to scenarios in which cell death is directly driven by the autophagy machinery, often due to excessive or dysregulated autophagic activity that leads to critical loss of cellular components. Conversely, AMCD denotes cases in which autophagy modulates or intersects with other dominant cell death pathways, such as apoptosis, necrosis, or pyroptosis, without being the primary cause of death [73]. For example, studies found that autophagy‐dependent ferroptosis via ferritinophagy and lipophagy is increasingly validated in cancer pathogenesis and treatment, which is a classical unique AMCD [74, 75, 76].

At the molecular level, autophagy is orchestrated through a series of highly regulated steps involving numerous signaling pathways and autophagy‐related genes (ATGs). The AMP‐activated protein kinase (AMPK)–mechanistic target of rapamycin complex 1 (mTORC1) axis plays a central role in initiating autophagy by promoting the formation of isolation membranes. The Beclin‐1/VPS34 complex is then recruited to drive vesicle nucleation and expansion. c‐Jun N‐terminal kinase (JNK)‐mediated phosphorylation of BCL‐2 and BIM results in the dissociation of Beclin‐1, further enhancing VPS34 activity. Subsequent vesicle elongation is mediated by the ATG12–ATG5–ATG16L complex and the lipidation of microtubule‐associated protein 1A/1B‐light chain 3 (LC3), a process converting LC3‐I to the autophagosome‐integrated form LC3‐II. Upon completion, autophagosomes fuse with lysosomes to form autolysosomes, enabling cargo degradation and recycling. Multiple upstream signaling cascades also converge on autophagy regulation, including EGFR/Ras/MEK/ERK, JNK/c‐JUN, p38/MAPK, and Wnt/β‐catenin pathways, which integrate extracellular and intracellular cues to fine‐tune autophagic flux [77]. Together, these coordinated molecular events position autophagy as a critical regulator of cell survival and death under both physiological and pathological conditions.

Recent foundational studies have illuminated the intricate interplay between autophagy and several key molecular pathways involved in cancer progression. Among these, SIRTs have emerged as critical regulators of diverse forms of PCD, including apoptosis, necroptosis, and autophagy. SIRTs modulate autophagic processes by targeting essential components such as Beclin1, LC3, and ATG proteins, while also influencing cellular energy metabolism and stress response pathways [78, 79]. In multiple cancer models, including pancreatic ductal adenocarcinoma (PDAC), lung cancer, and endometrial cancer (EC), SIRTs ablation has been shown to inhibit tumor cell proliferation and invasion via autophagy and disrupting cellular homeostasis [80, 81, 82]. Meanwhile, in genetically engineered mouse models, deletion of SIRT1 impaired autophagosome maturation and reduced autophagic flux, which led to the development of premalignant lesions, such as intraepithelial neoplasia and similar early‑stage neoplasias in other tissues [83]. In addition, SIRT1 deficiency has been associated with diminished mitophagy via regulation of the PINK1/Parkin pathway in cancers, further contributing to aberrant cell survival and transformation [81, 84, 85]. These findings collectively suggest that SIRTs serve as molecular “gatekeepers” of autophagy, limiting early oncogenic events and supporting their role as potential therapeutic targets across a wide range of cancers.

Androgen receptor (AR) signaling is another critical regulator of cancer‐related autophagy, especially in PCa. Recent studies have reported that small extracellular vesicles derived from prostate stromal cells enhance the radioresistance of PCa cells through the AMPK‐activated autophagy pathway, with stromal cells secreting higher levels of IL‐8 compared with AR‐positive PCa cells, such as LNCaP [86]. Besides, AR‐related autophagy has been observed across various clinical stages of the disease. The TOMM20‐triggered autophagic degradation, which is positively correlated with AR expression, can facilitate the transformation to neuroendocrine PCa (NEPC) [87]. Additionally, the regulation of expression by YAP1 appears to be influenced by autophagy activity in castration‐resistant PCa (CRPC), an advanced stage of PCa that continues to progress despite hormone therapy [88]. Thus, autophagic signaling exerts an imperative role both in the early stages of tumorigenesis and the progression of PCa. The role of autophagy in PCa is complex, as it can function as a survival mechanism or contribute to cell death, depending on the cellular context [89]. The interplay between autophagy and other PCD pathways, such as resistance to anoikis induced by cell detachment from the extracellular matrix, is mediated by the CEMIP signaling pathway through protective autophagy [90, 91]. Understanding and modulating the intricate mechanisms of autophagy in cancers presents a promising avenue for therapeutic exploration.

### 2.4 Pyroptosis: Inflammatory Damage‐Triggered Cell Death

Pyroptosis, categorized alongside apoptosis and necroptosis as a form of PANoptosis, is an inflammatory PCD [92]. PANoptosis is characterized by its inflammatory nature and involves signaling complexes including ZBP1, caspase‐8, and RIPK1/3 [93, 94]. The interconnectedness within PANoptosis arises from the initiation of these processes through some common receptors (e.g., PRRs) and their reliance on the cleavage functions of caspases. In contrast, pyroptosis is specifically defined by its dependence on caspase‐1 and the formation of gasdermin pores in the plasma membrane, which are essential for its inflammatory effects [95]. In the canonical inflammasome pathway, both pathogen‐associated molecular patterns (PAMPs) and damage‐associated molecular patterns (DAMPs) stimulate intracellular signaling molecules, leading to the assembly of inflammasomes composed of procaspase‐1. This complex activates caspase‐1, which along with caspase‐4/5, cleaves gasdermin D (GSDMD), resulting in pore formation and the release of proinflammatory cytokines such as interleukin‐1 beta (IL‐1β) and interleukin‐18 (IL‐18) that trigger subsequent cell lysis and execute pyroptosis. Besides, caspase‐11 can induce pyroptosis independent of caspase‐1 in a noncanonical pathway [96]. Dysregulation of pyroptosis has been implicated in various pathological conditions, including autoimmune diseases, inflammatory diseases, and cancers. Understanding the molecular mechanisms of pyroptosis is vital for developing targeted therapies.

The degree of pyroptotic activity varies markedly across cancer types and stages. A pan‐cancer analysis identified differential expression of 17 pyroptosis‐related genes, with higher expression levels associated with enhanced immune infiltration in “hot” tumors and poorer pyroptosis in “cold” tumors [97]. In gastric cancer, patients with low pyroptosis risk scores exhibited increased antitumor immune infiltration and better responses to neoadjuvant immunotherapy [98]. Similarly, in urological carcinomas, stratification based on pyroptosis gene signatures revealed subtypes with significant prognostic and immunologic differences [99, 100]. The pyroptosis cascade is modulated by several regulators, including caspases (especially caspase‐1/4/5/11), gasdermins (GSDMD, gasdermin E [GSDME]), and upstream inflammasomes. Dysregulation often involves genetic or epigenetic silencing of key components. For instance, USP48 was shown to stabilize GSDME, promoting pyroptosis and enhancing immunotherapy efficacy, suggesting its potential as a therapeutic target [101]. Additionally, noncoding RNAs and epigenetic mechanisms (e.g., DNA methylation) were found to regulate pyroptosis‐related gene expression, further influencing tumor progression and immune responsiveness [102].

Pyroptosis predominantly affects phagocytic cells like macrophages and has been implicated in cancer progression and treatment. The compound C10 was found to initiate pyroptosis in PCa by activating caspase‐3 and the PKCδ/JNK pathway, leading to GSDME cleavage and concurrent apoptosis and pyroptosis in PCa cells [103]. Hypermethylation of the GSDME promoter and reduced GSDME expression in PCa tissues are linked to a diminished response to PARP inhibitors [104]. Furthermore, GSDME‐mediated pyroptosis can be modulated by CDC20, which promotes GSDME ubiquitination [105]. Resistance to apoptosis may reduce pyroptosis sensitivity, and increased methionine levels are identified as antipyroptotic in “persister” cells, which exhibit a proliferative advantage under pyroptotic conditions [106]. Other anti‐inflammatory factors, including canopy FGF signaling regulator 3 (CNPY3) and the NOD‐like receptor family pyrin domain containing 3–caspase‐1–ASC complexes, have been shown to induce pyroptosis as an antitumor mechanism in cancers [107, 108]. The growing evidence for the antitumor effects of pyroptosis positions the targeting of pyroptotic or combined PANoptotic cell death as a promising therapeutic approach for cancer.

### 2.5 Entosis: Adhesion‐Driven Cellular Cannibalism

Entosis is a nonapoptotic cell death phenomenon in which one cell engulfs another, leading to internalization and subsequent lysosomal degradation [109]. The molecular mechanisms underlying entosis are centered around cell‐cell adhesion and actomyosin contractility, both of which are regulated by the RhoA–Rho‐associated protein kinase (ROCK) signaling pathway. This pathway is activated in response to various triggers, including loss of matrix adhesion, expression of E‐cadherin, and metabolic stress. The process begins with the formation of cell adhesions between the prospective host cell and the cell designated for internalization, primarily mediated by cadherin molecules. Following adhesion, actomyosin contractility driven by RhoA and ROCK, facilitates the internalization of the engulfed cell. The differential mechanical deformability between the two cells serves as the driving force for entosis [110]. Additional regulatory mechanisms involve AMPK, stress kinases such as JNK and p38, oncogenes such as KRas and c‐Myc, and tumor protein p53 (TP53). In cancers, entosis has been observed and is believed to promote disease progression by enabling more aggressive cells to internalize and eliminate aggressive counterparts [111]. Understanding the molecular mechanisms of entosis is important for developing targeted therapies, as this form of cell death is implicated in cancer progression and may present distinct vulnerabilities that can be exploited therapeutically.

Entosis can be induced by multiple intrinsic and extrinsic stressors, including matrix detachment, glucose starvation, mitotic arrest, and chemotherapeutic drugs like paclitaxel [112, 113]. In breast, colorectal, and pancreatic cancers, entosis occurs more frequently under conditions that favor cellular stress and competitive interactions among tumor cells. For instance, pancreatic tumors show a higher prevalence of entotic CICs in liver metastases compared with primary tumors, suggesting that entosis may be selectively activated during cancer dissemination [114]. The initiation and progression of entosis depend on actin‐myosin contraction in the internalizing cell, driven by Rho–ROCK signaling and modulated by cellular adhesion molecules like E‐cadherins and P‐cadherins [111]. Loss of RND3/RhoE in hepatocellular carcinoma enhances entosis through LAMP1 upregulation, implicating lysosomal activity in both initiation and degradation stages of the process [115]. Calcium signaling via Orai1 channels and cytoskeletal regulators like SEPTIN also contribute to the contractility and engulfment mechanics of entosis [116].

Entosis has paradoxical roles in tumor progression. On one hand, it promotes tumor suppression by killing genetically aberrant or detachment‐prone cells. On the other hand, it fosters tumor evolution by promoting aneuploidy, enhancing nutrient scavenging, and enabling survival under stress [117]. In PDAC, entosis is linked to the formation of aggressive subclones marked by NET1 overexpression, which drives enhanced proliferation, metastasis, and resistance to anoikis [24]. Similar associations have been observed in breast and colorectal cancers, where high CIC frequency correlates with poor prognosis and metastatic potential [118, 119]. Wen et al. [120] found that the AR can enhance entosis in CRPC via the Rho/ROCK pathway. Although the role of AR in cell death regulation is recognized, the specific process of entosis in tumor cells is not fully understood. Entosis may serve as a survival mechanism in some tumor cells under pro‐oxidant anticancer treatments [121]. In metastatic PCa, resistance to oxidative stress was accompanied by an increase in average cell size and polyploidization due to entosis, which facilitated the long‐term survival of cancer cells [122]. Entosis also has implications for cancer treatment. Liu et al. [123] showed that the tyrosine kinase inhibitor (TKI) nintedanib promotes entosis via the CDC42/ROCK pathway, and combining abiraterone with the Plk1 inhibitor onvansertib induced mitotic cancer cell death and entosis in abiraterone‐resistant metastatic CRPC mouse models independently of AR signaling. The research informed a phase II clinical trial (NCT03414034) and highlights the potential for manipulating entotic cell death in cancer therapy [124].

### 2.6 Ferroptosis: Fe‐Dependent Cell Death via Overwhelming Peroxides

**FIGURE 2:**
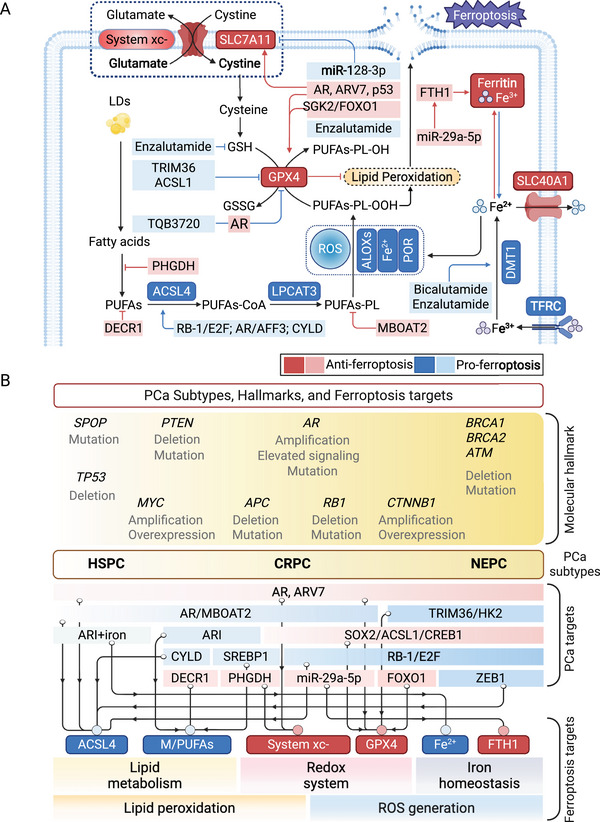
Ferroptosis pathway and cancer subtype‐related targets in PCa. (A) Canonical ferroptosis pathway and representative targets or drugs reported in PCa. The transporter system xc^−^ facilitates the entry of cystine into cells while simultaneously exporting glutamate. Inside the cells, cystine is converted to cysteine, which is utilized to produce GSH. GPX4 can convert lipid hydroperoxides into lipid alcohols, which makes the GSH–GPX4 system a crucial part in protecting cells against ferroptosis. A variety of proteins, including TFRC, SLC40A1, and components of ferritin (FTH1 and FTL), regulate ferroptosis by managing iron homeostasis. The synthesis of fatty acids, mediated by ACAC or the release of fatty acids through lipophagy, leads to an increase in intracellular free fatty acids, which in turn drives ferroptosis. ACSL4 and LPCAT3 enhance the integration of PUFAs into phospholipids, creating PUFAs‐PL that are susceptible to oxidation triggered by free radicals and mediated by ALOXs. Red and blue blocks represent anti‐ and proferroptosis factors, respectively. (B) Hallmarks of PCa subtypes and related regulators in ferroptosis‐targeted therapy. The hallmarks of PCa and targets in ferroptosis are shown in parallel with their corresponding clinical subtypes, including HSPC, CRPC, and NEPC. The regulatory targets are then assigned to the core processes of ferroptosis, including lipid peroxidation and ROS generation. Red and blue blocks represent anti‐ and proferroptosis factors, respectively. Targets specifically reported in PCa include AR, MBOAT2, TRIM36, SGK2/FOXO1, miR‐29a‐5p, and so on. *Abbreviations*: TFRC: transferrin receptor; SLC40A1: solute carrier family 40 member 1; ACAC: acetyl‐CoA carboxylase; FTL: ferritin light chain; PUFAs‐PL: PUFA‐enriched phospholipids; ALOXs: 5‐lipoxygenases. HSPC: hormone‐sensitive prostate cancer; CRPC: castration‐resistant prostate cancer; NEPC: neuroendocrine prostate cancer. The plot was created by BioRender.

Ferroptosis, a form of PCD characterized by Fe‐dependent lipid peroxidation, was first identified by Stockwell et al. [125]. Unlike apoptosis or necrosis, ferroptosis is characterized by the accumulation of lethal lipid peroxides, a process tightly regulated by the interplay between redox homeostasis, Fe metabolism, and membrane lipid composition. The initiation of ferroptosis hinges on an imbalance between proferroptotic processes and cellular antioxidant defenses. On the proferroptotic side, the biosynthesis and peroxidation of polyunsaturated fatty acid‐containing phospholipids (PUFA‐PLs) are key events, orchestrated by enzymes such as long‐chain acyl‐CoA synthetase 4 (ACSL4) and lysophosphatidylcholine acyltransferase 3 (LPCAT3). Concurrently, Fe‐dependent mitochondrial metabolism contributes to ROS generation and lipid peroxidation. In contrast, ferroptosis is counteracted by a suite of protective mechanisms, including the incorporation of monounsaturated fatty acids into phospholipids and both glutathione peroxidase 4 (GPX4)‐dependent and independent antioxidant systems, which function to detoxify lipid hydroperoxides [126]. When the oxidative burden surpasses the capacity of these defense systems, lipid peroxides accumulate and initiate membrane damage in an Fe‐catalyzed reaction, culminating in ferroptosis. Pharmacologically, this pathway can be manipulated using ferroptosis inducers (FINs). For example, erastin induces ferroptosis by inhibiting the cystine/glutamate antiporter system xc^−^, thereby depleting intracellular glutathione (GSH). The clinically applied kinase inhibitor sorafenib also induces ferroptosis via suppression of solute carrier family 7 member 11 (SLC7A11), the most important component of system xc^−^ [127]. Growing evidence implicates ferroptosis in the pathogenesis of cancer, neurodegenerative disorders, and other diseases, and ferroptosis‐related pathways are increasingly recognized as promising therapeutic targets in cancer, highlighting the potential for exploiting ferroptosis modulation in precision medicine approaches. Given the complicated disease conditions during PCa progression [128], the role of ferroptosis in PCa is provided to better comprehend this type of PCD (Figure 2).

Tumor cells with high Fe dependence, such as in TNBC and pancreatic cancer, exhibit heightened sensitivity to ferroptotic triggers [21]. Ferroptosis can be induced by pharmacological agents (e.g., erastin, RSL3), radiation, and cytokines like IFN‐γ and TGF‐β [129]. Transcription factors such as NF‐E2‐related factor‐2 (Nrf2) promote resistance to ferroptosis by upregulating antioxidant responses, while tumor suppressors like p53 can either enhance or inhibit ferroptosis depending on cellular context [21]. Additionally, tumor microenvironmental factors, such as lactate, glutamate, and immune cells, also modulate ferroptosis sensitivity [130]. Ferroptosis suppressor protein 1 (FSP1), previously known as AIFM2, provides a GPX4‐independent defense by regenerating reduced coenzyme Q10 (CoQ10), a lipophilic antioxidant, via NAD(P)H. FSP1 can prevent ferroptosis even in GPX4‐deficient cells and contributes to resistance in certain tumor types [131]. Induction of ferroptosis can eliminate mesenchymal and drug‐resistant cancer cells, offering a strategy against apoptosis‐resistant tumors. Paradoxically, ferroptosis‐induced inflammation may lead to immunosuppression in some contexts, thereby promoting tumor progression [21].

Hangauer et al. [132] revealed that patient‐derived cancer organoids with mesenchymal traits linked to neuroendocrine transformation (NET) were sensitive to GPX4 inhibition. Meantime, Viswanathan's work highlighted that lipid peroxidase pathway inhibition is prevalent in therapy‐resistant cancers, suggesting GPX4 and lipid peroxidation as therapeutic targets to combat drug resistance [133]. Subsequent studies identified regulators of GPX4 in PCa, such as serum/glucocorticoid regulated kinase 2 (SGK2), which can suppress ferroptosis and promote PCa metastasis by inhibiting FOXO1 effects on GPX4 [134]. More recent studies indicate that the GPX4‐mediated antiferroptosis effect can be suppressed by tripartite motif 36 (TRIM36) [135], low‐dose antimony treatment [136], or AR inhibition [137], which reverses the NET process and has demonstrated effectiveness in treating cancers. These studies have delineated the role of GPX4 as a crucial regulator of ferroptosis and a potential therapeutic target in cancers, with GPX4 inhibition showing promise, particularly in counteracting tumor growth, therapy resistance, and phenotype differentiation. In addition to GPX4, our latest study revealed that the activation of PLK1/TP53–SAT1 axis targeted by ezetimibe‐engineered compound obviously induced ferroptosis and suppressed tumor growth and invasion in PCa [138]. Another contemporary study also reported the ferroptosis‐mediated cancer suppression by the combinational treatment of ezetimibe and PARP inhibitor in TNBC [139].

In the lipid metabolism‐associated processes of ferroptosis, it has been widely recognized that the accumulation of PUFAs in the membrane determines the cellular sensitivity to peroxidation and the outcome of ferroptosis. The peroxidation propensity PUFAs surpasses that of monounsaturated and saturated fatty acids. Enzymes such as ACSL4 and LPCAT3, implicated in various cancers, activate and integrate PUFAs into cellular membranes to drive ferroptosis [140]. Among the limited studies, enzalutamide treatment has been shown to increase PUFA content and ferroptosis susceptibility in PCa cells by enhancing lipid uptake [141]. High ACSL4 and LPCAT3 expression enhances ferroptosis susceptibility in TNBC, hepatocellular carcinoma, and renal cell carcinoma through lipid reprogramming [142, 143, 144]. Castillo et al. [145] reported that the ACSL4 inhibitor PRGL493 suppresses cell growth and sensitizes them to chemotherapy and hormone treatment. Thus, an expanding body of evidence has confirmed the role of ACSL4‐induced ferroptosis in regulating tumor biology in a variety of circumstances. Further research is anticipated to explore this as a potential therapeutic direction.

### 2.7 Cuproptosis: Accumulative Cu‐Induced Cell Death

Cu, an essential micronutrient, is tightly regulated by proteins such as Cu transport protein 1 (CTR1) and six‐transmembrane epithelial antigen of the prostate (STEAP), with dysregulation implicated in diseases like Wilson's disease and cancer [146, 147]. In 2022, Tsvetkov et al. [3] identified a novel form of PCD triggered by intracellular Cu overload, which they termed cuproptosis. Unlike previously characterized PCD forms, cuproptosis operates through a unique mechanism and is resistant to inhibitors of apoptosis, necroptosis, ferroptosis, and other classical pathways. Only Cu chelators were effective in suppressing this Cu‐induced cytotoxicity, highlighting its mechanistic distinction [3]. Mechanistically, cuproptosis is initiated by the interaction of Cu ions with lipoylated components of the tricarboxylic acid (TCA) cycle, leading to protein aggregation and cellular stress. The Cu ionophore elesclomol (ES) facilitates the transport of Cu(II) to the mitochondria, where ferredoxin 1 (FDX1) reduces it to Cu(I). Accumulated Cu(I) interacts with lipoylated dihydrolipoamide S‐acetyltransferase (DLAT), inducing protein oligomerization and aggregation. Simultaneously, Cu accumulation destabilizes Fe–sulfur (Fe–S) cluster‐containing proteins, contributing to mitochondrial dysfunction and oxidative stress [148]. The defining hallmark of cuproptosis is the Cu‐dependent aggregation of lipoylated mitochondrial enzymes alongside the loss of Fe–S cluster proteins. This dual disruption ultimately leads to metabolic collapse and cell death. Aberrant Cu accumulation and its downstream effects have been linked to a range of pathological conditions, including tumorigenesis, suggesting cuproptosis as a potential target for therapeutic intervention in cancer and other diseases [149].

Cu's role in cancer cell death has a history dating to the 1980s, with disulfiram and ES as key Cu ionophores that induce melanoma cell death [150, 151]. Studies have implicated mitochondrial dysfunction and ROS in this process. However, applying the ROS inhibitor N‐acetylcysteine showed that ROS may not be the sole cause of Cu cytotoxicity [152]. Wen et al. [153] demonstrated that inducing cuproptosis can enhance the chemotherapeutic response of prostate tumors to docetaxel by inhibiting autophagy via the DLAT/mTOR pathway, which showed the connectivity between cuproptosis and other forms of cell death. Cuproptosis susceptibility is tightly linked to mitochondrial metabolism and Cu homeostasis. Tumors with high oxidative phosphorylation activity, such as clear‐cell renal cell carcinoma and PCa, exhibit greater sensitivity to cuproptosis‐inducing agents [154, 155]. In contrast, cancer stem cells (CSCs) in TNBC resist cuproptosis due to hypoxia‐mediated downregulation of FDX1, although this can be overcome by nanomaterial‐assisted photothermal therapy [156]. Cuproptosis also reshapes the tumor immune microenvironment. In colorectal and bladder cancers, cuproptosis‐associated gene signatures correlate with distinct immune infiltration patterns and predict response to immune checkpoint blockade [157, 158]. Furthermore, cuproptosis can enhance CD8⁺ T cell cytotoxicity by reducing PD‐L1 expression and suppressing Wnt/β‐catenin signaling in microsatellite‐stable colon cancer, overcoming immune resistance [159, 160].

Cuproptosis‐based therapies hold promise, especially in tumors resistant to apoptosis or ferroptosis. Predictive cuproptosis gene signatures have been developed for gastric, ovarian, and BCas to guide prognosis and drug response prediction [161, 162, 163]. Cu‐based nanomaterials have emerged as potent delivery systems to enhance tumor‐specific cuproptosis while minimizing systemic toxicity [164]. The AR antagonists also showed efficacy in inducing cuproptosis in treating cancers. Gao et al. [165] found that enzalutamide treatment enhanced the susceptibility of CRPC cells to cuproptosis by increasing their mitochondrial dependency. This vulnerability is specifically reversed using the Cu chelator tetrathiomolybdate, which binds to Cu ions and mitigates the cellular stress induced by Cu overload [165]. Despite these findings, the limited foundational research on cuproptosis in cancers constrains our understanding of its regulatory mechanisms and therapeutic potential, underscoring the need for further in‐depth studies.

### 2.8 Disulfidptosis: Unique Cell Death by Glucose Starvation in Cancer Cells

Disulfidptosis is a recently characterized form of PCD that occurs under conditions of glucose deprivation or impaired glucose uptake, specifically in cells with elevated expression of the cystine/glutamate antiporter subunit SLC7A11. This unique vulnerability was investigated by Liu et al. [5], who sought to clarify the mechanisms underlying rapid cell death in SLC7A11^high^ cancer cells. Under glucose‐starved conditions, SLC7A11^high^ cells experience excessive cystine uptake, which is intracellularly reduced to cysteine, a reaction that consumes large amounts of NADPH. The resultant NADPH depletion impairs the cell's redox balance, leading to the accumulation of disulfides and the induction of disulfide stress. Such stress activates the Rac–WRC–Arp2/3 signaling pathway, contributing to the formation of aberrant disulfide bonds in actin cytoskeleton proteins. This results in the collapse of F‐actin and detachment from the plasma membrane, ultimately culminating in cell death. The process is distinct from well‐known forms of cell death, such as apoptosis, necroptosis, and ferroptosis [5]. Its selective dependence on metabolic and redox imbalances in SLC7A11^high^ cancer cells underscores its potential as a therapeutic target, particularly in tumors with dysregulated cystine metabolism and high oxidative stress.

SLC7A11, also known as xCT, is a crucial member of the cystine transporter solute carrier family. Overexpression of SLC7A11 is commonly observed in cancer cells, where it facilitates cystine uptake, thereby protecting these cells from oxidative stress and ROS‐mediated cell death, including ferroptosis. Consequently, SLC7A11 has been regarded as a significant regulator that facilitates tumor survival and progression [166]. Nevertheless, a contemporaneous study found that varying levels of SLC7A11 overexpression led to distinctly different outcomes for cancer cells. Alongside research on disulfidptosis, these findings further elucidate the complex roles of SLC7A11 overexpression in cancer [167]. According to Liu et al., the premise of manipulating overexpressed SLC7A11 as a driver of disulfidptosis is glucose starvation, which occurs in conditions of the hypoxic TME, or as a result of targeted therapies that inhibit glucose uptake or utilization, such as glucose transporter (GLUT) inhibitors [5]. Glucose starvation is an emerging hallmark of cell survival‐related metabolic switches in cancer and may serve as a promising indicator of cellular sensitivity to various forms of cell death [169, 170]. Furthermore, ER stress has been identified as both a consequence and modulator of disulfidptosis, where its inhibition enhances cell death under glucose limitation [171]. Multiomics analyses reveal that disulfidptosis regulators are widely dysregulated across cancers due to DNA methylation changes and copy number variations, influencing tumor progression and therapeutic responsiveness [172].

Strategies to induce disulfidptosis, such as combining GLUT inhibitors with ER stress modulators, have shown antitumor effects in vitro and in vivo [171]. Glycogen synthase 1 (GYS1) has also emerged as a therapeutic target to trigger disulfidptosis in TNBC [173]. To evaluate the potential of disulfidptosis in cancer therapy, Zhao et al. [174] integrated multiomics and pan‐cancer approaches to construct a molecular landmark of tumor disulfidptosis across over 30 cancer types. Their experimental validation proved that disulfidptosis‐related genes could be effectively targeted by various antitumor agents [174]. The crosstalk between disulfidptosis and ferroptosis, particularly involving the SLC7A11 and cystine system, has also been further elucidated in cancers [175, 176]. Disulfidptosis activity influences the tumor immune microenvironment (TME), with certain disulfidptosis subtypes showing enhanced CD8⁺ T cell infiltration and improved immunotherapy response, notably in renal and cervical cancers [177, 178]. With more in‐depth research into the mechanisms of disulfidptosis, novel insights could be gained to utilize disulfidptosis as a therapeutic target in addressing various challenges associated with PCa.

## 3 Connectivity Between PCD

**FIGURE 3:**
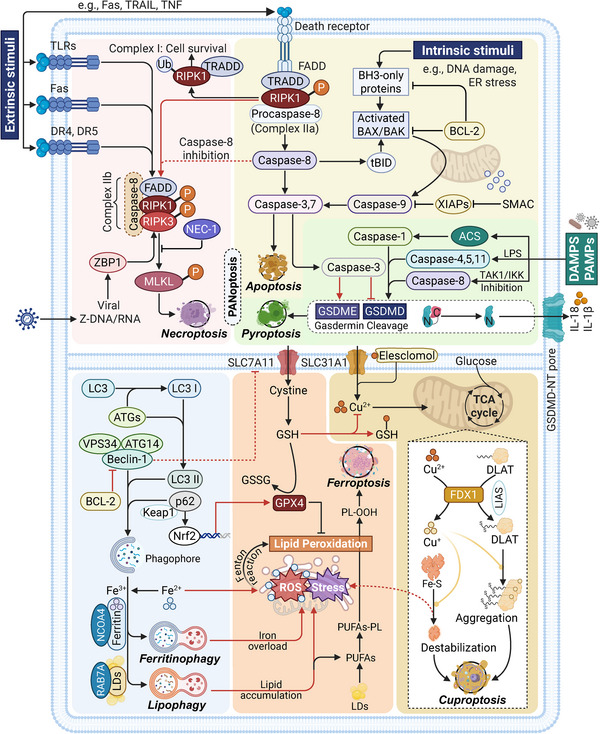
Crosstalk between different PCD forms and their core mechanisms. This network illustration introduces 6 major forms of PCD indicated in the review, including apoptosis, necroptosis, pyroptosis, autophagy, ferroptosis, and cuproptosis. Apoptosis, necroptosis, and pyroptosis are closely linked and constitute the PANoptosis based on sharing molecular drivers such as RIPK1 and caspases family. The formation of different RIPK1–TRADD complexes (I, IIa, IIb) determines the PCD type and cell fate, including survival, apoptosis, and necroptosis. Caspase‐8 is the common protein that participates in these three PCDs, making up PANoptosomes along with ZBP1 and ACS in PANoptosis. In autophagy, the driving factor beclin‐1 has been recognized as a novel BH3‐only protein that can be intrinsically suppressed by BCL‐2, making up its association with apoptosis [183]. Next, autophagy tightly interacts with ferroptosis, especially in the form of ferritinophagy and lipophagy, which provide fundamental substrate iron and lipids to reinforce the core process (Fenton reaction and lipid peroxidation) of ferroptosis. The p62–Nrf2 pathway is another reverse regulatory approach on ferroptosis due to its transcriptional role on GPX4, as reported by the latest studies [139, 369, 370]. The GSH metabolism, involved in the ferroptosis redox system, can inhibit cuproptosis initiation by chelating copper(II). Finally, the destabilization of the Fe–S complex enhances the ROS level, strengthening the core process of autophagic, ferroptotic, and cuproptotic cell death. Blocks of different colors represent different PCDs. The red lines indicate the association between different PCDs. Abbreviations: ACS: apoptosis‐associated speck‐like protein, SMAC: second mitochondria‐derived activator of caspases, XIAP: X‐linked inhibitor of apoptosis protein, Ub: ubiquitination, P: phosphorylation, PL‐OOH: phospholipid hydroperoxides, LDs: lipid droplets, NCOA4: nuclear receptor coactivator 4, RAB7A: RAS‐related in Brain 7A. The plot was created by BioRender.

Cell death pathways are essential for maintaining cellular homeostasis and defending against diseases. Despite their distinct morphological and molecular characteristics, these pathways share commonalities and exhibit differences in their mechanisms, roles, and regulation (Table 1). We also summarize the molecular connections between major PCDs to illustrate their intrinsic crosstalk, as shown in Figure 3.

**TABLE 1:** Established markers, therapeutic targets, and biological hallmarks of various PCDs.

PCD type	Markers and targets	Biological hallmark
Apoptosis	BCLs, p53, Fas, TNFR1, DR4/5, IAPs, APAF1, caspase‐3/7/8/9	Cellular stress and death receptor initiation mediated activation of effector caspases
Necroptosis	Nec‐1, Fas, TNFR1, DR4/5, TLR, RIPK1/3, MLKL, ZBP1	Extracellular signal mediated complex formation consisted of RIPKs and MLKL
Autophagy	ATGs, AMPK, mTOR, LC3, Beclin‐1, BCL‐2, BIM	Maturation of the autophagosome and fusion with a lysosome to form an autolysosome
Pyroptosis	GSDMD, caspase‐1/4/5/8, IL‐1β, IL‐18	Caspase‐induced GSDMD cleavage, subsequent pore formation and proinflammatory cytokines secretion
Entosis	RhoA, ROCK, AMPK, JNK, p38, DR4/5	Cell engulfment triggered by detachment and contractile activity
Ferroptosis	GPX4, SLC7A11/xCT, ACSL4, LPCAT3, TFR1, PUFAs	Imbalance of redox system induces accumulation of lipid peroxides
Cuproptosis	FDX1, DLAT, Fe–S, CTR1, STEAP	Aggregation of lipoylated mitochondrial enzymes and decrease of Fe–S protein
Disulfidptosis	SLC7A11/xCT, GLUT, Rac–WRC–Arp2/3, SLC3A2	NADPH depletion and disulfides accumulation under glucose starvation and SLC7A11 overexpression

Abbreviations: IAPs, inhibitor of apoptosis proteins; APAF1, apoptotic protease‐activating factor 1; Rac, Ras‐related C3 botulinum toxin substrate; WRC, WAVE regulatory complex; Arp2/3, actin‐related protein 2/3 complex; SLC3A2, solute carrier family 3 member 2.

Initiation through membrane receptor activation is a common mechanism in many PCDs. Extrinsic apoptosis and necroptosis are both initiated by the activation of cell surface death receptors, such as TNFR1 and Fas, which trigger intracellular signaling cascades involving adaptor proteins and kinases. Besides, pyroptosis also shares PRRs‐driven activation of signaling transduction with alternative necroptosis that responds to PAMPs and DAMPs [179]. It should be noted that caspase families, a group of cysteine proteases capable of cleaving substrates after aspartic acid residues, are extensively involved in multiple PCD forms such as apoptosis, necroptosis, and pyroptosis [180]. Notably, the differential activation of caspases distinguishes the fate of cell death in various contexts. Caspase‐1 is a crucial initiator of pyroptosis; however, it does not participate in apoptosis. In contrast, the downstream caspases involved in apoptosis, such as caspase‐3 and caspase‐7, inhibit pyroptosis by inactivating GSDMD. Similarly, caspase‐8 plays an essential role in facilitating both apoptosis and pyroptosis, while also serving as a critical inhibitor of necroptosis [6]. Furthermore, the interaction between BCL‐2 and beclin‐1, two key regulators in PCD, establishes a crucial molecular link between apoptosis and autophagy. Abundant studies have demonstrated that BCL‐2 directly binds to beclin‐1, and the abundance of this interaction significantly influences the level of cellular autophagic activity. Disrupting the BCL‐2/beclin‐1 binding has emerged as an effective strategy to enhance autophagy [181, 182]. Further mechanistic investigations suggest that beclin‐1 itself may function as a BH3‐only protein, inherently susceptible to inhibition by BCL‐2, thereby underscoring its dual regulatory potential in PCD [183].

Another hallmark of PCD connection is the redox system imbalance and metabolism dysregulation, which are common causes of ion‐mediated cell death, including ferroptosis, cuproptosis, and disulfidptosis. Ferroptosis and cuproptosis are classic metal ion‐dependent forms of PCD in which excessive levels of divalent Fe and Cu lead to lipid peroxidation and the aggregation of lipoylated proteins, respectively, resulting in cell death associated with toxicant overload. Disulfidptosis, driven by disorders in glucose metabolism and NADPH depletion, culminates in cell death related to the accumulation of disulfide bonds. The intrinsic connection between these forms of PCD, characterized by redox system imbalance and cellular metabolic disruption, can yield synergistic therapeutic effects in cancer treatment. For instance, two clinically utilized FINs, sorafenib and erastin, can significantly mediate cuproptosis by increasing the expression of FDX1 and reducing GSH synthesis in liver cancer [184]. Similarly, PPARγ antagonists can activate both ferroptosis and disulfidptosis by upregulating heme oxygenase 1 (HMOX1) and SLC7A11 [185]. Increasing evidence shows that SLC7A11/xCT, one of the vital regulators of intracellular antioxidants, has become a focal point for the synchronous regulation of these metabolic disorder‐associated PCDs [186]. Moreover, cellular stress responses and ROS‐associated events are extensively implicated in various PCD‐related biological processes, including apoptotic and autophagic mitochondrial dysfunction, ferroptotic lipid hydroperoxides, and cuproptotic TCA cycle disorders [187, 188]. Finally, there is substantial evidence of crosstalk in molecular signaling between cell death pathways, where they can be simultaneously modulated by certain upstream regulators, such as RCN1, the SIRT family, mTOR, PI3K/Akt, and MAPK signaling, as mentioned above [9, 189]. Understanding these commonalities and differences is crucial for the development of targeted therapies and for deciphering the complex interplay between cell death pathways in disease pathogenesis.

## 4 Targeting PCD in Cancer Treatment

Understanding PCD mechanisms across diverse malignancies is critical for developing precise and effective cancer therapies. Contemporary oncologic management integrates surgery, RT, cytotoxic chemotherapy, immunotherapy, and hormone therapies that leverage distinct PCD pathways to counter tumor progression and resistance. Adjuvant RT and chemotherapy remain standard in intermediate‐ and high‐risk tumors, inducing cell death primarily via DNA damage‐triggered apoptosis and mitotic catastrophe [190]. In the past decade, immunotherapies, particularly ICIs (e.g., anti‐PD‐1/PD‐L1, CTLA‐4), have reactivated T cell‐mediated cytotoxicity by enhancing extrinsic apoptotic signaling across melanoma, lung, and renal cancers [191]. In parallel, targeted therapies directed at oncogenic drivers (e.g., EGFR, ALK, BRAF, PARP) demonstrate that inhibiting kinase signaling or DNA repair can induce both apoptotic and nonapoptotic PCD forms, often synergizing with conventional agents [192].

The emergence of novel PCD modalities, such as ferroptosis, pyroptosis, and cuproptosis, has broadened the therapeutic landscape. Ferroptosis is being investigated using GPX4 inhibitors and agents that disrupt GSH homeostasis, particularly in therapy‐resistant tumors [193]. Combination approaches based on metal ions have shown synergistic antitumor efficacy in preclinical models [194]. Collectively, these findings underscore the translational potential of modulating diverse PCD networks to overcome therapeutic resistance and advance next‐generation cancer treatments (Table 2).

**TABLE 2:** Reported targets and mechanisms of the PCD‐based treatment in cancers.

PCD type	Treatment	Target/Mechanism	Cancer type	Refs
Apoptosis	Castration + ADT	Antiandrogen, TGF‐β activation	Prostate cancer	[195]
	CD24–CAR NK	BCL‐2 signaling blockade	Testis, prostate, renal, bladder cancer	[196]
	Quercetin	Dual inhibition of Wnt3a/β‐catenin and Akt/NF‐κB pathway; modulation of MAPK/ERK and JAK/STAT3 pathways, etc.	Breast, colorectal, liver cancer, glioblastoma, osteosarcoma	[197, 198, 199, 200]
	NKG2D–CAR T/NK + IL‐7/IL‐21	Activation of T cells and NK cells	Lung, prostate cancer, and glioblastoma	[201, 202, 203]
	Ultrasound + docetaxel	Increased cytotoxicity by ultrasound exposure	Liver, lung, prostate cancer	[204, 205, 206]
	Delta‐tocotrienol (δ‐TT)	ER stress and ROS production through JNK/p38 activation; p16/CDK4/cyclin D1 pathway activation, etc.	Bladder, nasopharyngeal, ovarian, pancreatic, prostate cancer	[207, 208, 209, 210, 211]
	Selenium	ROS/p53/Bax‐mediated TRAIL activation; STAT3/JNK pathway activation	Colorectal, lung, ovarian, prostate cancer	[212, 213, 214, 215]
	Ophiopogonin D′	RIPK1 and Bim activation; disrupting RGS4 overexpression; p53 activation	Thyroid, colon, prostate cancer	[216, 217, 218]
	Curcumin	p38 MAPK activation; IRON‐chelating via TfR1/IRP1 activation; ROS induction, etc.	Breast, colorectal, liver lung, prostate cancer	[219, 220, 221, 222]
	Celastrol	ROS induction; NF‐κB/Akt inhibition and JNK activation; PRDX inhibition, etc.	Gastric, esophageal, liver, colorectal, prostate cancer	[223, 224, 225, 226, 227]
	Corosolic acid	IRE‐1/JNK activation; JAK2/STAT3 activation	Breast, pancreatic, prostate cancer	[228, 229, 230]
Necroptosis	Shikonin	RIP3/p62/Keap1 complex; T cells infiltration; RIP1/RIP3 activation, etc.	Breast, bladder, bile duct, prostate cancer	[231, 232, 233, 234]
	SMAC mimetics	TNF‐α/TNFR1 signalling; MLKL expression	Bladder cancer, lymphoma	[235, 236]
	δ‐TT	MLKL expression and translocation	Prostate cancer	[237]
	Ophiopogonin D'	RIPK1/MLKL signaling blockade	Prostate cancer	[216]
	Selenium	ROS induced‐RIP1 regulation	Prostate cancer	[238]
	Photodynamic therapy	Increased oxidative stress	Liver, ovarian, prostate cancer	[239, 240, 241, 242]
Autophagy	δ‐TT	ER stress through pJNK/p38 activation; release of mitochondrial ROS	Liver, prostate cancer	[207, 243]
	Chloroquine/hydroxychloroquine	Autophagosome–lysosome fusions break and discontinue	Breast, colorectal, liver cancer	[244, 245]
	Curcumin	Iron deprivation; mitochondrial membrane rupture	Colorectal, lung, prostate cancer	[219, 246, 247]
	Celastrol	ER stress; ROS‐mediated mitochondrial dysfunction	Breast, colorectal, prostate cancer	[248, 249, 250, 251, 252]
	High‐dose DHT	AR/miR‐493‐3p/PARK2 pathway activation	Prostate cancer	[253, 254]
	Photodynamic therapy	Increased oxidative stress; ROS/JNK pathway activation	Breast, colorectal, prostate cancer	[255, 256]
Ferroptosis	Erastin/RSL3	GPX4/SLC7A11 inhibition	Breast, bladder, gastric, kidney, prostate cancer	[257, 258, 259, 260, 261]
	Sorafenib	SLC7A11 inhibition; oxidative stress	Liver, cervical, kidney cancer	[262, 263, 264, 265]
	Iron supplementation	Enhanced oxidative stress and lipid peroxidation	Breast, prostate cancer, melanoma, glioblastoma	[266, 267, 268]
	Statins	Mevalonate pathway downregulation and GPX4 inhibition; antitumor immunity activation; autophagy dependence, etc.	Breast, gastric, lung cancer	[269, 270, 271, 272]
	Curcumin	GPX4 and SLC7A11 inhibition; HCAR1/MCT1/SREBP1 pathway, etc.	Breast, liver, lung, ovarian cancer	[247, 273, 274, 275]
	Ezetimibe	NPC1L1 inhibition; PLK1/TP53/SAT1 activation	Breast, prostate cancer	[138, 139]
	Flubendazole	P53‐mediated SLC7A11 inhibition; downregulation of xCT and GPX4	Breast, prostate cancer, glioblastoma	[276, 277, 278]
	CAR‐NK cells and nanoplatform	HER2 and PSMA targeting; GPX4/SLC7A11 inhibition	Breast, prostate cancer	[279, 280, 281]
	Nanoparticles (NP)	Excessive iron oxide supplementation; PUFAs peroxidation; GPX4 inhibition	Colorectal, prostate cancer, lymphoma, acute myeloid leukemia	[282, 283, 284, 285]
	Polyphyllin I	Nrf2/FTH1 suppression; xCT/GPX4 inhibition; ERK/DNMT1/ACSL4 pathway activation	Gastric, liver, prostate cancer	[286, 287, 288]
	Sanguinarine	GPX4 inhibition; ROS/BACH1/HMOX1 pathway activation	Cervical, lung, prostate cancer	[289, 290, 291]
Cuproptosis	ES–Cu	Excessive copper accumulation; DLAT/mTOR pathway activation	Breast, colorectal, liver, prostate cancer	[153, 184, 292, 293]
	Copper complex NPs	Excessive copper‐induced cytotoxicity; immune responses	Breast, colorectal, live, pancreatic, prostate cancer	[294, 295, 296, 297, 298, 299, 300]
Pyroptosis	Gambogic acid	ROS/P53/mitochondria/caspase‐3 activation; SIRT1‐mediated CNPY3 localization, etc.	Breast, colorectal, ovarian, prostate cancer	[108, 301, 302, 303]
	Chemotherapy	Caspase‐1 and caspase‐3 signaling activation	Breast, colorectal, esophageal, lung cancer	[104, 304, 305, 306, 307]
	Methionine	GSDME degradation; preservation of plasma membrane integrity	Esophageal, liver, lung, prostate cancer, head and neck squamous cell carcinoma	[106, 308]
Entosis	Ultraviolet radiation (UV)	JNK and p38 stress‐activated kinase signaling	Breast cancer	[309]
	Nintedanib	CDC42/ROCK signaling activation	Prostate cancer	[123]
	Onvansertib	Plk1 inhibition	Prostate cancer	[124]
Disulfidptosis	Natural products	SLC7A11/NADPH signaling targeting	Breast, liver cancer	[310]
	NU1025	DCS3 subtype sensitivity	Clear cell renal cell carcinoma	[310]

Abbreviations: CDK4, cyclin‐dependent kinase 4; NF‐κB, nuclear factor kappa‐light‐chain‐enhancer of activated B cells; PRDX, peroxiredoxin; IRE‐1, inositol‐requiring enzyme 1; Keap1, Kelch‐like ECH‐associated protein 1; SREBP1, sterol regulatory element‐binding protein 1; PSMA, prostate‐specific membrane antigen; DCS3, differential screening‐selected gene aberrative in neuroblastoma 3.

### 4.1 Targeting Cell Death in Chemotherapy

Chemotherapy remains a foundational approach to cancer treatment, with increasing emphasis on its ability to engage PCD pathways to overcome resistance and improve outcomes. Traditional chemotherapeutic agents often act by inducing apoptosis, but novel strategies now exploit alternative cell death mechanisms such as necroptosis, ferroptosis, and autophagy to circumvent apoptosis resistance, a common trait in various tumors. Apoptosis is the most widely studied mechanism of chemotherapy‐induced PCD. Many cytotoxic drugs trigger apoptosis through intrinsic or extrinsic pathways, and resistance to chemotherapy often stems from dysregulation of key apoptotic regulators like BCL‐2, p53, and caspases [311]. Small molecules such as navitoclax and obatoclax that target antiapoptotic Bcl‐2 family proteins are under investigation for enhancing chemosensitivity [312]. Necroptosis may offer advantages in apoptosis‐resistant tumors such as glioblastoma and pancreatic cancer. Agents like shikonin have been explored for their necroptosis‐inducing properties to enhance chemotherapy efficacy in vitro [313]. Chemotherapeutics such as doxorubicin and paclitaxel can modulate autophagy‐related pathways to augment cytotoxicity in resistant BCa [314]. Besides, traditional Chinese medicine (TCM) has emerged as a therapeutic contender, with our study showing Qingdai decoction inhibits CRPC cell growth by dampening the AKT pathway, which is crucial for the occurrence of autophagy and apoptosis [315]. These results indicate that supplementary agents may help overcome chemotherapeutic resistance in refractory cancer patients.

Beyond apoptosis‐centered PCD forms, ion‐dependent cell death modalities such as ferroptosis and cuproptosis have garnered interest for their role in sensitizing resistant cancer cells. Chemotherapy agents such as cisplatin and doxorubicin have been shown to promote ferroptosis by depleting GSH and inhibiting GPX4, especially in breast, colorectal, and hepatocellular carcinomas [194, 316]. While still in early investigation stages, cuproptosis inducers have demonstrated the capacity to overcome resistance in preclinical models of various solid tumors when combined with standard chemotherapy [317]. Importantly, ferroptosis and cuproptosis exhibit significant molecular crosstalk. Recent evidence suggests that mitochondrial Cu overload can sensitize cells to ferroptosis via enhanced ROS production and GPX4 inhibition [318]. Combinatorial regimens of cuproptosis and ferroptosis inducers are now being tested with promising potential to overcome resistance mechanisms in TNBC and lung adenocarcinoma [319, 320].

Moreover, chemotherapy may indirectly contribute to immune evasion by inducing PD‐L1 overexpression in the TME through NF‐κB activation, as shown in ovarian and gastric cancers. This highlights the need for combining chemotherapy with ICIs to sustain antitumor immunity [321, 322]. Finally, nanomedicine represents a transformative direction in cancer chemotherapy by enabling the targeted delivery of agents that induce specific PCD mechanisms, reducing systemic toxicity while enhancing tumor specificity [317].

### 4.2 Targeting Cell Death in Immunotherapy

Immunotherapy has revolutionized oncology by harnessing the body's immune system to recognize and eradicate tumor cells. Among these strategies, ICIs, particularly PD‐1/PD‐L1 axis blockade, have demonstrated long‐lasting clinical efficacy across multiple cancers. Recent advances underscore that their therapeutic benefit is not only derived from immune activation, but also through the modulation and induction of diverse forms of PCD. Traditionally, the success of PD‐1/PD‐L1 blockade has been attributed to the reactivation of cytotoxic CD8⁺ T cells, leading to apoptosis of tumor cells. However, recent insights suggest that immune checkpoint inhibition also promotes nonapoptotic cell death forms [14]. Immune activation may also trigger pyroptosis and necroptosis, which release proinflammatory mediators like IL‐1β and DAMPs. In parallel, pyroptosis facilitates an antitumor immune microenvironment and boosts the efficacy of immunotherapy. Inhibition of CDC20 potentiates antitumor immunity by triggering GSDME‐mediated pyroptosis in tumor [105]. These processes amplify immune cell recruitment and potentiate ICI efficacy. Combinatorial induction of these death forms, termed PANoptosis, has been proposed to overcome immunotherapy resistance [323].

Chimeric antigen receptor (CAR)‐based therapies, including CAR‐T and CAR‐NK cells, are being optimized to overcome immunosuppressive barriers in solid tumors. CAR‐T therapies have shown efficacy in hematologic malignancies, but challenges such as cytokine release syndrome, T cell exhaustion, and immunosuppressive TME limit their effectiveness in solid cancers [324]. Innovations such as IL‐7‐expressing CAR‐T cells have demonstrated improved persistence and reduced apoptosis of CD8⁺ T cells by modulating BCL‐2 signaling pathways, enhancing their cytotoxic durability [14]. CAR‐NK platforms represent a promising next‐generation approach, combining innate immunity with engineered antigen specificity. Compared with CAR‐T cells, CAR‐NK therapies offer advantages such as lower toxicity, lack of GvHD, and the ability to function independently of MHC presentation [325, 326]. CAR‐NK cells targeting tumor‐specific proteins such as CD24 and prostate‐specific membrane antigen (PSMA) have shown robust induction of apoptosis in tumor cells via Fas–FasL signaling and perforin/granzyme B pathways [327].

Ferroptosis is tightly linked to immune modulation. CD8⁺ T cells, activated by PD‐1/PD‐L1 blockade, enhance ferroptosis via IFN‐γ‐mediated suppression of SLC7A11 and GPX4, leading to oxidative damage in tumor cells [328, 329]. Moreover, ferroptosis itself stimulates immunogenic cell death (ICD), releasing DAMPs (e.g., HMGB1, ATP) that promote dendritic cell maturation and T‐cell priming. Preclinical studies show that nanoplatforms combining ferroptosis inducers with anti‐PD‐L1 antibodies synergize to enhance antitumor responses [330, 331, 332]. Clinical relevance is further supported by findings that genes regulating ferroptosis, such as Decr2, determine tumor sensitivity to immunotherapy [329]. Several nano‐enabled immunotherapy platforms have demonstrated that inducing cuproptosis can reshape the TME and potentiate PD‐L1 blockade, even in resistant cancers [333, 334]. Additionally, dual ferroptosis‐cuproptosis platforms, such as AuBiCu‐PEG NPs, have been used to amplify RT and immunotherapy responses, overcome PD‐L1 upregulation, and enhance systemic immunity [335]. More recently, evidence suggests that CAR‐NK cells may also induce ferroptosis through the release of IFN‐γ and ROS, particularly when targeting PSMA‐positive solid tumors. For instance, PSMA is highly expressed in PCa cells, and the emerging PSMA‐targeted immune strategy offers the advantages of high specificity and efficiency in treating PCa. Combined treatment with anti‐PSMA CAR‐NK cells and anti‐PD‐L1 monoclonal antibody enhances the effectiveness of antitumor therapy against CRPC. The CAR‐NK92MI cells against PSMA‐targeted polypeptide selectively killed PSMA‐positive C4‐2 cells through IFN‐γ‐mediated ferroptosis [279, 336]. This mode of cell death can bypass classical apoptosis resistance and contribute to antitumor immunity [337]. Additionally, memory‐like CAR‐NK cells, induced by IL‐12/15/18 activation, exhibit enhanced persistence and killing capacity against resistant cancer types, suggesting a potential role in durable tumor control [338].

### 4.3 Targeting Cell Death in RT

RT remains a cornerstone of cancer treatment and is increasingly understood not only as a DNA‐damaging therapy but also as a modulator of PCD. Its therapeutic efficacy largely depends on its ability to activate intrinsic and extrinsic death pathways in tumor cells. Apoptosis is the primary mechanism through which RT exerts its cytotoxic effects. Radiation‐induced DNA damage activates p53‐related pathways that culminate in MOMP and caspase‐8 activation [339]. However, mutations in apoptosis regulators such as TP53 or BCL2 often confer resistance to apoptosis, prompting the need to target alternative PCD pathways. Radiation can trigger pyroptosis through caspase‐1 or ‐3 activation, resulting in enhanced antitumor immunity via cytokine release and immune cell recruitment [340]. Harnessing pyroptosis may not only potentiate tumor clearance but also mitigate resistance observed in apoptosis‐impaired cancers. It is noteworthy that RT‐induced apoptosis in immune cells may cause clinical side effects of the treatment [341]. RT also modulates autophagy, which serves a protective function in PCa under radiation therapy. Overexpression of LC3A provides a functional survival mechanism for PCa cells in response to RT by activating autophagy [342]. While autophagy may initially protect cancer cells by clearing damaged organelles, excessive autophagy can lead to type II PCD. Combining RT with autophagy inhibitors (e.g., chloroquine) has shown promise in enhancing radiosensitivity in glioblastoma and pancreatic cancer [343].

RT increases ROS and depletes GSH, making cancer cells susceptible to metal ion‐dependent PCD. The key regulators of ferroptosis, such as GPX4 and SLC7A11, are downregulated by ionizing radiation, sensitizing tumor cells to ferroptosis and promoting tumor regression [194]. It has also been shown to enhance cellular Cu uptake and oxidative stress, thereby potentially priming cells for cuproptosis [318]. Besides, emerging findings indicate that ferroptosis‐related molecules may act as markers for predicting radiosensitivity and PCD in PCa [344, 345]. Moreover, these pathways contribute to RT‐induced ICD. The release of oxidized lipids, mitochondrial DAMPs, and proinflammatory cytokines from ferroptotic and cuproptotic cells can enhance dendritic cell activation and T‐cell recruitment, suggesting a role for metal‐dependent death in the abscopal effect and immune priming [346]. RT not only kills tumor cells directly but also acts as an immunogenic trigger, shaping the TME through PCD‐induced immunostimulatory effects. This is exemplified by the enhanced efficacy of combining RT with PD‐1/PD‐L1 ICIs. Several studies have shown that RT upregulates PD‐L1 expression on tumor cells, and its blockade synergizes with radiation to enhance tumor antigen presentation and T‐cell activation [347, 348].

### 4.4 Targeting Cell Death in Targeted Therapy

Targeted therapy has revolutionized cancer treatment by selectively inhibiting molecular pathways essential for tumor survival and proliferation [190]. Beyond cytostatic effects, increasing evidence shows that many targeted agents exert therapeutic efficacy by engaging PCD pathways, including apoptosis, ferroptosis, cuproptosis, and emerging modalities such as disulfidptosis and entosis. TKIs targeting EGFR, ALK, or KRAS not only inhibit mitogenic signaling but also activate apoptosis and pyroptosis, as shown by gasdermin‐mediated membrane permeabilization following caspase‐3 activation [349]. Venetoclax, a BCL‐2 inhibitor approved for chronic lymphocytic leukemia, reinstates the mitochondrial apoptosis pathway by antagonizing antiapoptotic proteins [350].

In recent years, targeting ferroptosis has represented a significant focus of small‐molecule drug discovery. Compounds like erastin, RSL3, sulfasalazine, and sorafenib deplete intracellular GSH or inhibit GPX4, leading to ROS accumulation and ferroptotic death [351]. Novel molecules such as MESA (a morpholine derivative) specifically target the Nrf2 pathway, sensitizing prostate and ovarian cancer cells to ferroptosis via GPX4 downregulation and Fe overload [352]. Other compounds like Compound 8 inhibit the xCT transporter (SLC7A11), depleting GSH and triggering ferroptosis, and show enhanced activity compared with classical inducers [353]. Small‐molecule ferroptosis inducers are now being engineered to specifically target CSCs, with evidence that certain ROS‐inducing compounds exhibit higher toxicity against mesenchymal BCa subpopulations [354]. Moreover, strategies that transition ferroptosis to apoptosis by transcriptional reprogramming or small molecule cocktails are being explored to enhance therapeutic outcomes and avoid resistance [355]. Adding to this repertoire, disulfidptosis, a newly identified death form triggered by disulfide stress under glucose starvation, is being targeted by small molecules that inhibit GLUT1 or exacerbate ER stress [356], offering unique therapeutic entry points, especially in tumors with high SLC7A11 expression. Taken together, these examples underscore that modern targeted therapy is no longer limited to growth inhibition. Rather, it increasingly seeks to orchestrate selective PCD engagement using pharmacologically refined small molecules, providing new therapeutic angles to tackle apoptosis resistance, metabolic rewiring, and tumor heterogeneity.

### 4.5 Targeting Cell Death in Hormone Therapy

Endocrine or hormone therapy plays a central role in the treatment of hormone‐driven malignancies such as BCa, EC, and PCa, where therapeutic strategies typically aim to suppress ER or AR signaling. However, treatment resistance frequently arises, which is often mediated by impaired cell death responses. Increasing evidence reveals that manipulation of PCD can sensitize hormone‐refractory tumors and augment therapeutic efficacy. In androgen deprivation therapy (ADT)‐resistant PCa, which is CRPC, targeting lipid metabolism regulators such as PHGDH, DECR1, and MBOAT1/2 restores ferroptosis susceptibility and enhances antitumor responses. Inhibition of these enzymes leads to Fe‐dependent lipid peroxidation and cell death even in the presence of AR signaling [357], suggesting a promising route for next‐generation ADT. The impact of autophagy on ADT is a subject of ongoing investigation. In the context of ADT‐induced energy deficiency, AMPK is activated and triggers autophagy, which provides an apparent survival advantage for PCa cells [358]. Despite the dual nature of autophagy, its stimulation by ADT tends to negatively affect tumor growth. Inhibition of autophagy has been proposed to enhance the effectiveness of combined androgen ablation and chemotherapy [359], and it is also considered a strategy to boost the efficacy of abiraterone in CRPC [360]. Recent findings suggest that high‐dose androgen dihydrotestosterone (DHT) can induce autophagy and cell death in enzalutamide‐resistant PCa [253]. SIRT1, a vital regulator of autophagy in PCa, might be a potential parallel target of ADT therapy. Its inhibition can amplify the effects of enzalutamide and abiraterone on PCa [83].

**FIGURE 4:**
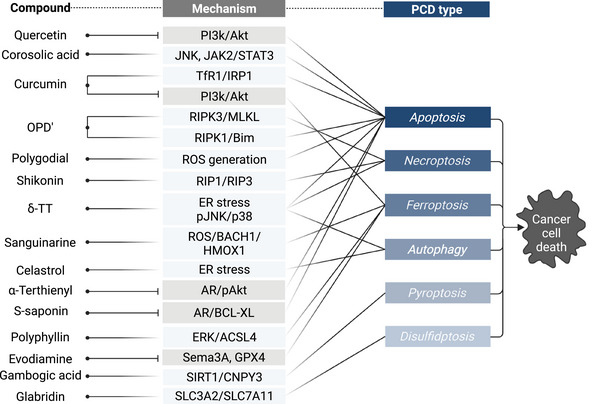
Representative natural compounds that target PCD for the treatment of cancer. Numerous natural products and bioactive components derived from TCM have been reported to exhibit antitumor effects by inducing PCD. Here, we summarize representative compounds targeting specific PCD modalities, along with their molecular mechanisms, to demonstrate the potential of PCD modulation in natural product‐based cancer therapy. *Abbreviation*: TCM: traditional Chinese medicine.

In hormone receptor‐positive BCa, autophagy appears to mediate resistance to antiestrogen agents like tamoxifen or aromatase inhibitors. Metronomic chemotherapy combined with endocrine therapy (e.g., cyclophosphamide and letrozole) has shown improved outcomes, in part by reactivating apoptotic pathways, as well as inducing autophagy‐related stress that promotes cell vulnerability [361]. The GPX4‐independent regulation of ferroptosis by sex hormone receptors (e.g., ER, AR) demonstrates its significant potential in influencing hormone‐dependent cancers [357]. In EC, a prototypical hormone‐driven tumor, recent studies underscore the relevance of cell death regulation in the efficacy of endocrine therapy. Gonadotropin‐releasing hormone (GnRH) analogs, including agonists and antagonists, have been shown to directly inhibit EC cell proliferation via activation of apoptotic pathways, such as MAPK–GADD45α signaling, caspase‐3 cleavage, and PKCδ–p53/p21 cascade activation [362, 363]. Furthermore, ferroptosis and cuproptosis have emerged as promising avenues in EC research. Transcriptomic and clinical analyses have identified ferroptosis‐related gene signatures and long noncoding RNAs that predict prognosis and immune landscape, supporting the feasibility of ferroptosis‐guided precision endocrine therapy [364, 365]. Finally, natural products are also emerging approaches for cancer treatment that target different PCDs (Figure 4).

For the clinical application, several of the mentioned compounds have already undergone clinical trials in solid tumors [124, 366, 367]. As mechanistic research into PCD‐based treatments expands and clinical practices increase, the landscape of cancer therapy is poised for significant evolution. We summarized representative clinical trials in Table 3, and more comprehensive information can be referred to the online clinical research study database “Clinicaltrials.gov.”

**TABLE 3:** Representative clinical trials of anticancer treatment targeting PCD.

Drug/treatment	PCD type	Cancer type	NCT number
Quercetin + dasatinib	Apoptosis	Breast cancer Prostate cancer Multiple myeloma	NCT06355037 NCT01538316 NCT01912820 NCT06940297
NKG2D‐CAR T cells	Apoptosis	Colorectal cancer Gastric cancer Liver cancer Prostate cancer Sarcoma	NCT04107142 NCT05248048 NCT06087341 NCT05382377
δ‐TT/γ‐TT	Apoptosis, autophagy	Breast cancer Prostate cancer Pancreatic cancer	NCT00844792 NCT01571921 NCT00985777
Selenium	Apoptosis, necroptosis	Lung cancer Bladder cancer Prostate cancer	NCT00008385 NCT00729287 NCT00030901 NCT00022165 NCT01155791
Curcumin	Apoptosis, autophagy Ferroptosis	Breast cancer Colorectal cancer Pancreatic cancer Pediatric cancer Prostate cancer Head and neck cancer	NCT03980509 NCT00027495 NCT00094445 NCT06044142 NCT02064673 NCT04208334
BI2536	Necroptosis	Breast cancer Endometrial cancer Lung cancer Pancreatic cancer Prostate cancer Lymphoma	NCT00526149 NCT00376623 NCT00710710 NCT00706498 NCT00243087
Photodynamic therapy	Necroptosis, autophagy	Bile duct cancer Cervical cancer Esophageal cancer Oral cancer Prostate cancer	NCT00005067 NCT02304770 NCT01755013 NCT03638622 NCT01043016
SMAC mimetics	Necroptosis	Lung cancer Multiple myeloma	NCT03270176 NCT01955434 NCT03111992
Iron nanoparticles	Ferroptosis	Breast cancer Cervical cancer Colorectal cancer Liver cancer Lymphoma	NCT06048367 NCT04682847 NCT06048367 NCT01815333
Statins	Ferroptosis	Breast cancer Pancreatic cancer Prostate cancer	NCT00816244 NCT03454529 NCT06241352 NCT01759836
Elesclomol (STA‐4783)	Cuproptosis	Epithelial cancer Kidney cancer Lung cancer Prostate cancer	NCT00888615 NCT00088088 NCT00808418
Onvansertib	Entosis	Colorectal cancer Pancreatic cancer Prostate cancer Acute myeloid leukemia	NCT05593328 NCT04752696 NCT03414034 NCT03303339
Nintedanib (BIBF 1120)	Entosis	Cervical cancer Lung cancer Ovarian cancer Prostate cancer	NCT02009579 NCT01948141 NCT00710762 NCT02182219

*Note*: Data were acquired from NIH‐ClinicalTrials.gov (https://clinicaltrials.gov/).

## 5 Conclusion and Perspective

Therapeutic approaches targeting PCD have quickly gained traction as a promising direction in cancer treatment. Traditional strategies like chemotherapy, RT, and some targeted therapies primarily aim to induce apoptosis. However, resistance to apoptosis, especially in advanced or treatment‐resistant tumors, remains a major hurdle that often leads to poor clinical outcomes. To address this, newer forms of PCD such as ferroptosis, pyroptosis, and cuproptosis are being explored as alternative methods to eliminate cancer cells and bypass resistance pathways. Recent studies emphasize the potential benefits of incorporating these nonapoptotic death pathways into cancer therapy. For instance, inducers of ferroptosis have demonstrated effectiveness in preclinical models of therapy‐resistant cancers, including breast, prostate, and pancreatic malignancies. Likewise, triggering PANoptosis could amplify both immune system responses and direct tumor cell killing [14, 17].

Despite these advances, significant challenges persist. These include risks related to unintended toxicity, variable responses depending on the tumor context, and a still‐evolving understanding of how different cell death mechanisms interact. Moving forward, it will be essential to map out the intricate relationships among various PCD pathways in the tumor. Combination strategies, like pairing ferroptosis activators with immune checkpoint blockade or targeting molecules that regulate multiple PCD types, might offer more robust outcomes while avoiding resistance loops. At the same time, leveraging specific metabolic or genetic traits unique to tumors will be key to ensuring precise and selective activation of cell death.

Building on the existing crosstalk among various forms of PCD, advanced CRISPR‐based technologies offer a powerful platform to identify key molecular mediators that orchestrate the interplay between different PCD pathways [368]. By strategically activating multiple PCD mechanisms, this approach holds promise in overcoming resistance to a single mode of cell death. Emerging technologies, such as single‐cell RNA sequencing and spatial transcriptomics, are likely to accelerate the discovery of biomarkers that can guide these approaches. Ultimately, the success of PCD‐centered therapies may rely on developing precision models that customize treatment based on the unique PCD profiles of individuals. As our knowledge base expands and more therapies progress from lab to clinic, these strategies hold the potential to reshape how we treat cancer, offering renewed hope in the fight against therapeutic resistance and improving long‐term patient outcomes.

## Author Contributions

Conceptualization: Fuwen Yuan. Writing – original draft: Yuang Wei and Fuwen Yuan. Writing – review and editing: William Hankey and Dongliang Xu. Supervision: Fuwen Yuan and Dongliang Xu. All authors have read and approved the final manuscript.

## Ethics Statement

The authors have nothing to report.

## Conflicts of Interest

The authors declare no conflicts of interest.

## Data Availability

The authors have nothing to report.
